# Brain Transport Profiles of Ginsenoside Rb_1_ by Glucose Transporter 1: *In Vitro* and *in Vivo*

**DOI:** 10.3389/fphar.2018.00398

**Published:** 2018-04-19

**Authors:** Yu-Zhu Wang, Qing Xu, Wei Wu, Ying Liu, Ying Jiang, Qing-Qing Cai, Qian-Zhou Lv, Xiao-Yu Li

**Affiliations:** Department of Pharmacy, Zhongshan Hospital, Fudan University, Shanghai, China

**Keywords:** carrier-mediated transporter, drug transport, blood–brain barrier, glucose transporter 1, ginsenoside Rb_1_

## Abstract

Ginsenoside Rb_1_ (Rb_1_) has been demonstrated its protection for central nervous system and is apparently highly distributed to the brain. The objective of this study was to characterize Rb_1_ transport at the blood–brain barrier (BBB) using primary cultured rat brain microvascular endothelial cells (rBMEC), an *in vitro* BBB model. The initial uptake velocity of Rb_1_ in rBMEC was temperature- and concentration-dependent, and was significantly reduced by phloretin, an inhibitor of GLUT1 transporter, but was independent of metabolic inhibitor. Furthermore, the transport of Rb_1_ into rBMEC was significantly diminished in the presence of natural substrate α-D-glucose, suggesting a facilitated transport of Rb_1_ via GLUT1 transporter. The impact of GLUT1 on the distribution of Rb_1_ between brain and plasma was studied experimentally in rats. Administration of phloretin (5 mg/kg, i.v.) to normal rats for consecutive 1 week before Rb_1_ (10 mg/kg, i.v.) at 0.5, 2, and 6 h did not alter Rb_1_ concentrations in plasma, but resulted in significant decreased brain concentrations of Rb_1_ compared to in the phloretin-untreated normal rats (489.6 ± 58.3 versus 105.1 ± 15.1 ng/g, 193.8 ± 11.1 versus 84.8 ± 4.1 ng/g, and 114.2 ± 24.0 versus 39.9 ± 4.9 ng/g, respectively). The expression of GLUT1 in the phloretin-treated group by western blotting analysis *in vitro* and *in vivo* experiments was significantly decreased, indicating that the decreased transport of Rb_1_ in brain was well related to the down-regulated function and level of GLUT1. Therefore, our *in vitro* and *in vivo* results indicate that the transport of Rb_1_ at the BBB is at least partly mediated by GLUT1 transporter.

## Introduction

The root of ginseng has been in widespread use for Chinese traditional medicine for millions of years. It has been suggested to have a varieties of pharmacological activities such as vasorelaxation, anti-inflammatory, antidiabetic, anticancer, antioxidation (Kang et al., 2006; Lu et al., 2009; Gao et al., 2010; Qi et al., 2010). The effects of ginseng are mostly attributed to a class of dammarane-type triterpene saponins known as ginsenosides. These ginsenosides are further classified according to their aglycone moieties as 20(S)-protopanaxadiol type (ginsenoside Rb_1_, Rb_2_, Rb_3_, Rc, and Rd) and 20(S)-protopanaxatriol (ginsenoside Re, Rg_1_, Rg_2_, and Rh_1_) (Liu et al., 2009). Ginsenoside Rb_1_ is one of prominent active constituents in the root of ginseng and accumulating evidence indicates that Rb_1_ possesses neuroprotection for central nervous system (CNS). Particularly, Rb_1_ was considered as a potent agent, which could protect brain from ischemia-reperfusion injury possibly by scavenging free radicals, preserving the structural integrity of the neurons, modulating the expression of apoptosis-associated proteins, inhibiting the expression of Beclin 1 and LC3 via activation of PI3K/Akt pathway, and upregulating levels of brain-derived neurotrophic factors (Jiang and Qian, 1995; Lim et al., 1997; Zhang et al., 2006; Liu et al., 2009; Gao et al., 2010; Lu et al., 2011).

Our previous study has determined pharmacokinetic and absolute bioavailability of Total Panax Notoginsenoside (TPNS) after intravenous administration of TPNS injection at the dosage of 10.0 mg/kg to rats. Results show that the largest content of Rb_1_ and its duration in brain tissue are up to about 1.4 μg/g and 24 h, whereas those of Rg_1_ are only about 0.2 μg/g and 1 h. Rb_1_ and Rg_1_ are present in the greatest amounts in TPNS, because the concentrations of Rb_1_ and Rg_1_ are in large amounts in rat plasma or excretory samples. The contents of Rb_1_ and Rg_1_ in TPNS are almostly equivalent, about 34 and 26%, respectively (Li et al., 2007). This phenomenon indicates that Rb_1_ is capable of crossing the blood–brain barrier (BBB) into brain and the specific distribution of Rb_1_ in rat brain has no obvious correlation with its high content in TPNS. As we all know, the BBB is consisted of brain microvascular endothelial cells (BMEC) working together with astrocytes and pericytes, in which the physical barrier developed by the endothelial tight junctions and the transporter barrier resulting from membrane transporters strictly control the entry of endogenous and exogenous substances from the circulating blood into the CNS, therefore playing an essential role in brain homeostasis and protecting the CNS against many toxic compounds (Abbott et al., 2010). The characteristics of the BBB include high electric impedance, low water conductivity, high reflection coefficient, and low diffusion permeability of water-soluble substances. Evidence from the above described indicates that the impenetrability of BBB constitutes a barrier to most polar molecules and macromolecules delivery to brain parenchyma (Cardoso et al., 2010). The polarity of Rb_1_ is high and its molecular weight is also large, about 1109.29. So we speculate that some transporters in BMEC membranes are possibly involved in the transport of Rb_1_ across the BBB. However, what transporter involved in the transport of Rb_1_ across the BBB has not been reported in the present study so far.

GLUT proteins are encoded by the SLC2 genes and belong to members of the major facilitator superfamily of membrane transporters, which primary function is to transport extracellular glucose from interstitial fluid across the cellular plasma membrane by a passive and facilitative transport process along with the downward gradient concentration of glucose (Shibuya et al., 2014). GLUT proteins consist of 14 members, and GLUT1 and GLUT3 are mostly expressed in the CNS (Mueckler and Thorens, 2013). The ginsenoside Rb_1_ possesses two glucose moieties at the C-3 and C-20 positions, respectively, so we hypothesize that GLUT1 or GLUT3 in BMEC membranes is involved in the transport of Rb_1_ across cell membranes.

Therefore, the aim of this study was to demonstrate the transport mechanism of Rb_1_ across the BBB into brain. First, we measured the uptake kinetics of Rb_1_ using rBMEC *in vitro* for the sake of obtaining evidence for active uptake of Rb_1_ into cells. We then further investigated the effects of multiple inhibitors of transporters on the uptake of Rb_1_ into rBMEC. Finally, the brain-to-plasma concentration ratio value of Rb_1_ (*K*_p_) in the phloretin-treated normal rats was measured using LC–MS/MS method for further obtaining evidence that transport of Rb_1_ across the BBB is actively facilitated by GLUT1 or GLUT3 transporter.

## Materials and Methods

### Materials

Ginsenoside Rb_1_ and digoxin (internal standard, IS) were purchased from the National Institute for the Control of Pharmaceutical and Biological Products (Beijing, China). Collagenase type II, bovine serum albumin (BSA), gelatin, phloretin, and D (+)-glucose were purchased from Sigma (Sigma-Aldrich Co. LLC., United States). Fetal bovine serum (FBS) was purchase from GIBCO (Invitrogen Corporation, United States). Dulbecco’s modified Eagle’s medium (DMEM-F12) was purchased from HyClone (Thermo Scientific, United States). Rabbit anti-GLUT1, rabbit anti-GLUT3, and goat anti-rabbit IgG (H+L) conjugated with the appropriate horseradish peroxidase were purchased from Abcam. All other chemicals and reagents were of analytical or LC–MS grade as appropriate.

### Animals

Male Sprague-Dawley rats (220–250 g) were supplied by Center of Experimental Animals, Shanghai First People’s Hospital Affiliated Shanghai Jiao Tong University. Animals were housed under a constant environment condition (temperature, 25 ± 1°C; humidity, 50 ± 5%) with free access to standard laboratory diet and water. Sprague-Dawley neonate rats of 10 days old were used. All the experiments were performed in accordance with China’s Guidelines for Care and Use of Laboratory Animals.

### Isolation and Primary Culture of rBMEC

Isolation and culture of rat brain microvessel endothelial cells were operated according to previous studies with some modifications (Liu et al., 2013). The isolated rBMEC was seeded in cultured flasks coated with 2% gelatin and cultured in culture medium. The culture medium consisted of DMEM/F12 supplemented with 20% FBS, 2 mM L-glutamine, heparin (50 mg/L), 100 IU/mL penicillin, 100 μg/mL streptomycin, and NaHCO_3_ (0.5 g/L). On the first day after seeding, the culture medium was replaced, after which the culture medium was replaced every 2 days. At 5–6 days, the rBMEC was transferred to 6-well plates coated with gelatin and cultured in an incubator with a saturated humidity at 37°C in 5% CO_2_ and 95% air. Transport studies were performed when cells reached 100% confluency. The endothelial phenotype of the rBMEC was identified by staining for the endothelial cell marker vWF using a confocal laser scanning microscope (Leica TCS SP8).

### Transport Studies

When rBMEC was grown to 100% confluency in gelatin-coated 6-well plates, uptake experiments were performed. Briefly, the cultured cell monolayer was washed three times with 1 mL of pH 7.4 Hanks’ balanced salt solution (HBSS). The cultured rBMEC was pre-incubated for 30 min in 1 mL HBSS at 37 or 4°C. After pre-incubation, the HBSS solution was removed by suction, and HBSS (1 mL) containing 60 μg/mL Rb_1_ was added to initiate uptake. Then the cells were incubated at 37 or 4°C at a set time (5, 10, 15, 30, 60, 120, and 240 min). After the end of the incubation, the incubation solution was aspirated and the monolayers were carefully washed three times with 1 mL ice-cold HBSS to terminate the uptake. Then, 0.5 mL ultrapure water was added to each incubated well, and the material in the wells was frozen (at -80°C)/thawed (at room temperature) repeatedly four times to lyse cells. After the solubilized, the monolayer supernatants were collected and 10 μL was injected into LC–MS/MS to determine Rb_1_ concentration. The protein contents of the cell monolayers were measured by the method of Bradford using BSA as the standard (Bradford, 1976). Uptake was expressed as the cell-to-medium ratio (μL/mg protein or μL/mg protein/min) and termed the volume of distribution (*V*_d_), obtained by dividing the uptake amount by the concentration of substrate in the transport medium. *V*_d_ is derived from the ratio of ng/mg protein to ng/μL buffer.

In order to assess the kinetic parameters, Rb_1_ uptake data (7.5 to 960 μg/mL for 120 min) were analyzed using Michaelis-Menten plots based on the following equation: $V=\frac{V_{\max}\times S}{K_{m}\times S}+P_{diff}\times S$, where *V* is the initial uptake rate of substrate (nmol/mg protein/min), *V*_max_ is the maximum uptake rate (nmol/mg protein/min), *S* is the concentration of Rb_1_ in the medium (μM), *K*_m_ is the Michaelis-Menten constant (μM), and *P*_diff_ is the non-saturable uptake clearance (μL/mg protein/min), respectively (Kitamura et al., 2014). The uptake data were fitted to the above equation by nonlinear least-squares regression analysis with Graphpad Prism 5.0 software.

### Transporter Inhibition Assays

Our study showed that the uptake of Rb_1_ by rBMEC was dependent on time, concentration, and temperature. A steady-state uptake occurred at 120 min. In the present study, the uptake at 120 min was selected to measure the effect of test agents on the transport characteristics of Rb_1_. Rb_1_ concentration was set to be 60 μg/mL. The rBMEC was seeded out in 6-well plates, allowed to adhere, and grown to confluency. To study the transport mechanisms, uptake of Rb_1_ was carried out in the presence of a metabolic energy inhibitor and a series of established transporter inhibitors. The impact of these inhibitors on Rb_1_ uptake by rBMEC was assessed at the steady state at 37°C. NaN_3_ (500 μM), probenecid (100 μM), CsA (25, 50, and 100 μM), or phloretin (5 μM) was added to inhibit different transport systems. In the inhibition study, to further assess the effect of phloretin on Rb_1_ uptake into rBMEC, the cells were incubated at 37°C at a set time (5, 15, 30, 60, and 120 min). Uptake was measured after incubation with Rb_1_ in the presence of phloretin at the concentration of 5 μM.

In the competition experiments, Rb_1_ uptake (30–960 μg/mL) was measured in the absence and presence of D-glucose (100 mM) which is a principal physiological substrate of GLUT1. Briefly, cells were grown in 6-well plates for 7 days before their medium was removed, pre-incubated with warm glucose-free Hanks’ solution for 30 min. Subsequently, samples and controls were treated as described transport studies, but this time in addition with 100 mM D-glucose to the various concentrations of Rb_1_ in glucose-free Hanks’ solution at 37°C for 120 min.

### Cell Viability Assessment

The cytotoxic effects of the test agents used in this study were assessed on confluent monolayers of cells in 96-well plates using a CCK-8 assay. Cells were subjected to a 200 μL/well of each drug in Hanks’ solution at the concentrations used in the experiments, followed by incubation for another 4 h. Then, 10 μL Cell Counting Kit-8 (CCK8, Dojindo) solution was added to each well. Plates were incubated for additional 4 h. Absorbance readings at 450 nm were obtained using a spectrophotometer (Thermo Varioskan) and expressed as percentage viability compared to control untreated cells.

### Effect of Phloretin on Distribution of Rb_1_ in Plasma and Brain of Normal Rats

Rats were randomly divided into two groups in 36 of each group. One group served as the control and another group were treated with phloretin. Dose of phloretin was set to be 5 mg/kg. Rb_1_ (10 mg/kg, *i.v.*) was administered to the experimental rats after *i.v.* administration of phloretin or saline for consecutive 1 week. Six rats from each group were selected and the blood samples were collected into heparinized Eppendorf tubes via the abdominal aorta at 0.5, 2, and 6 h after Rb_1_ administration. Then, brain samples were immediately collected. The plasma samples were obtained by centrifuging at 1000 × *g* for 10 min. Plasma and brain samples were frozen at -80°C until analysis.

### HPLC–MS/MS Method for Rapid Quantification of Rb_1_ in Cells, Plasma, and Brain

HPLC–MS/MS was composed by a Shimadzu LC-20A chromatographic system and an API 4000 mass spectrometer equipped with electrospray ionization (ESI) source system. MS/MS detection was performed on an API 4000 mass spectrometer using multiple reaction monitoring (MRM) mode by monitoring the fragmentation of *m*/*z* 1107.6 → 179.0 for Rb_1_ and *m*/*z* 779.4 → 345.2 for digoxin (IS). Chromatographic separations were carried out on a Shim-pack XD-ODS column (2.0 mm × 30 mm, 2.2 μm) with a Shim-pack GVD-ODS (2.0 mm × 5 mm, 4.6 μm) guard column (Shimadzu, Japan) at a flow rate of 0.28 mL/min using 10 mM acetic acid in water (phase A) and methanol (phase B) as mobile phase. A linear step gradient elution was performed as followed: phase B was increased from 45 to 90% within the first 3 min, and decreased to 45% within the next 3 min (total gradient time: 6 min). A 10 μL sample was injected into the system with the auto-sample conditioned at 4°C and column temperature maintained at 40°C.

The biological samples (100 μL) were placed in a 1.5 mL Eppendorf tube, and mixed with 10 μL IS solution (500 ng/mL) for 3 min by vortexing. The mixture was extracted with methanol (0.9 mL) by vortexing, and then centrifuged at 14,000 rpm for 5 min. The supernatant (0.8 mL) was transferred to a new 1.5 mL Eppendorf tube and evaporated to dryness under vacuum. The dried residue was reconstituted with 100 μL methanol, vortex-mixed for 30 s, and centrifuged at 14,000 rpm for 5 min. Finally, 10 μL of the supernatant liquid was immediately subjected to HPLC–MS/MS analysis. The system control and data analysis were performed by AB Sciex Analyst software (the software version: Analyst 1.5.1). Retention time for Rb_1_ was *t*_R_ = 3.80 ± 0.01 min and for IS digoxin *t*_R_ = 3.13 ± 0.01 min. The lowest limits of quantitation of Rb_1_ in cell lysate, plasma, and brain homogenate were 1, 2.5, and 1 ng/mL, respectively. The recoveries were higher than 90%; the relative standard deviations (SD) of intra-day and inter-day were lower than 10%.

### Western Blotting

Total proteins were extracted and 60 μg of each protein was separated on SDS-10% polyacrylamide gels. Proteins on the gel were transferred to PVDF membrane (Merck-Millipore). Blots were blocked with 5% skimmed milk dissolved in PBS containing 0.1% Tween-20 (PBST) for 2 h at room temperature. The membrane was incubated with primary antibody at 4°C overnight. Washing of the blots with PBST three times was followed by incubation with horseradish peroxidase-conjugated secondary antibody for 2 h at room temperature. After washing, the chemiluminescence signal was monitored using the ChemiDoc XRS+ system (Bio-Rad, Hercules, CA, United States). Densitometric semi-quantitative analysis of protein bands was carried out using Image J.

### Statistical Analysis

All data were expressed as mean ± SD. Statistical significance of difference among groups was evaluated by one-way of analysis of variance (ANOVA) and the Student’s *t*-test. A *P*-value of <0.05 indicated a statistical significance.

## Results

### Uptake Kinetics of Rb_1_ by rBMEC

**FIGURE 1:**
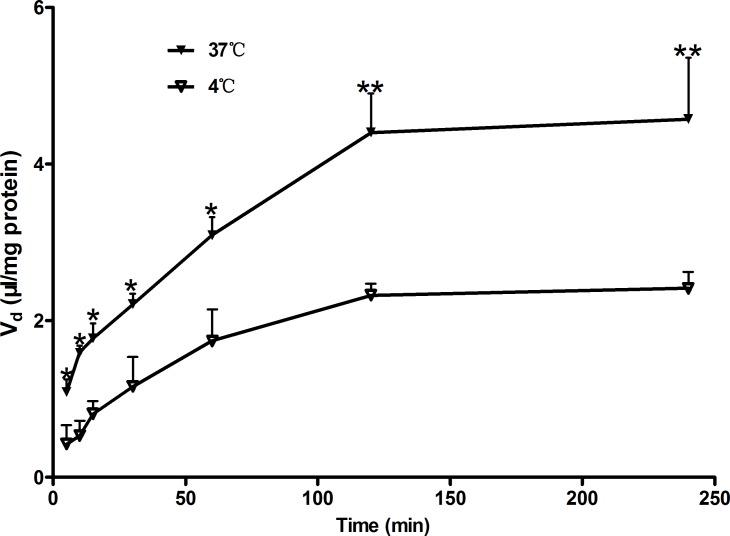
Time course of Rb_1_ uptake by rBMEC. Uptake of Rb_1_ (60 μg/mL) was measured at 37°C (black markers) and 4°C (white markers) with primary rBMEC cultured on 6-well plates for different times. Each point represents the mean ± SE (*n* = 3). Asterisks show a significant difference (^∗^*P* < 0.05 and ^∗∗^*P* < 0.01 versus white markers).

Uptake of Rb_1_ by rBMEC was evaluated at various intervals (5, 10, 15, 30, 60, 120, and 240 min) to obtain the optimal time for uptake studies. **Figure 1** shows the time course of the uptake of Rb_1_ by rBMEC at a concentration of 60 μg/mL at 37 and at 4°C. Rb_1_ uptake increased linearly with time until 120 min, and a plateau of accumulation was observed at 120–240 min. Accordingly, uptake at 120 min was used for subsequent kinetic and inhibition studies. However, in contrast, the uptake rate at 4°C was slow and the intracellular amounts of Rb_1_ were about two to threefold greater at 37°C than those at 4°C. This indicated transporters in rBMEC membranes were involved in the Rb_1_ transport.

**FIGURE 2:**
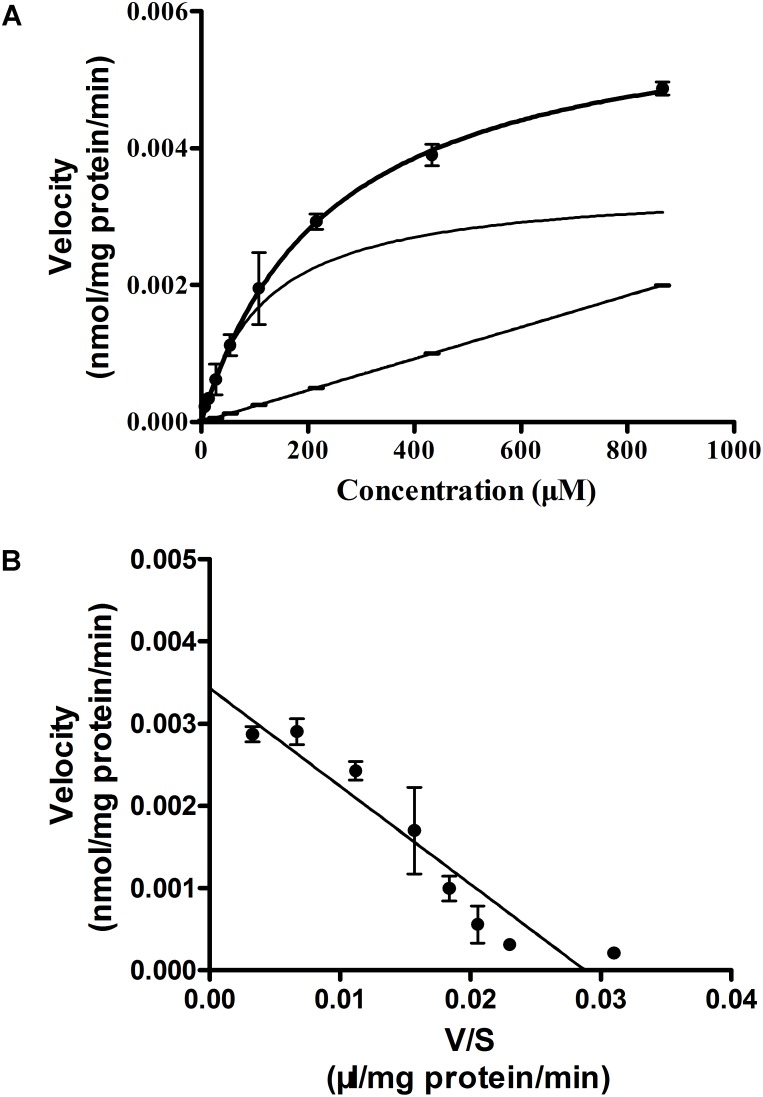
Concentration-dependence of Rb_1_ uptake **(A)** into rBMEC and the Eadie–Hofstee plot **(B)**. rBMEC was incubated in Rb_1_ solution at different concentration (7.5, 15, 30, 60, 120, 240, 480, and 960 μg/mL) at 37°C for 120 min. Cells were lysed and the intracellular amount of Rb_1_ was evaluated by LC–MS/MS. Each point represents the mean ± SE (*n* = 3). The solid curve, solid line, and curve represent estimated total, nonsaturable, and saturable uptakes, respectively **(A)**. *V* and *S* represent initial uptake velocity [nmol/(mg protein ⋅ min)] of the saturable component and concentration of Rb_1_ **(B)**.

To study the mechanism of Rb_1_ transport, uptake of Rb_1_ by rBMEC was examined at various concentrations (7.5–960 μg/mL) at steady state and the kinetics parameters (*K*_m_ and *V*_max_) were investigated by fitting the data to the *Michaelis-Menten equation*. As shown in **Figure 2A**, initial uptake of Rb_1_ showed concentration dependency. Kinetics analysis provided a *K*_m_ value of 119.2 ± 15.6 μM and a *V*_max_ of 0.0034 ± 0.0003 nmol/(mg protein ⋅ min). The Eadie–Hofsee plot for saturation uptake, fitted by subtraction of nonsaturable uptake from total uptake, approximately appeared a straight line (*R*^2^ = 0.9066), indicating involvement of a simple passive membrane diffusion (**Figure 2B**).

### Inhibition of Rb_1_ Uptake

**FIGURE 3:**
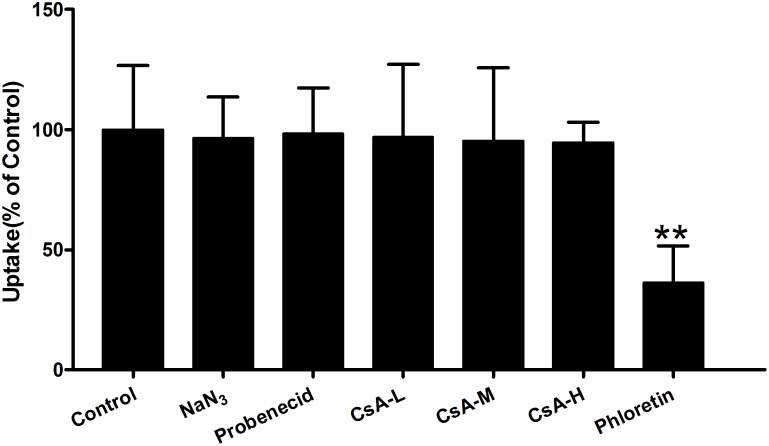
Effect of ATP-depletion and influx or efflux transporter inhibitors on Rb_1_ uptake into rBMEC. Rb_1_ uptake (60 μg/mL) was measured in the absence (control) or presence of sodium azide (NaN_3_, 500 μM), probenecid (100 μM), cytochalasin A (CsA, 25, 50, and 100 μM), and phloretin (5 μM) at 37°C for 120 min. Each column represents the mean ± SE (*n* = 3) with significant differences from control shown as ^∗∗^*P* < 0.01.

To clarify whether the accumulation of Rb_1_ in rBMEC was not only related to a simple passive diffusion, but to facilitated diffusion, we performed the effects of various inhibitors of transporters that facilitate the uptake of Rb_1_ into rBMEC. As shown in **Figure 3**, while no statistically significant differences were observed with inhibitors of the ABCB1 (P-gp, CsA) (Sun et al., 2006), organic anion-transporting polypeptides (OATPs, probenecid) (Jørgensen et al., 2007), and metabolic energy (NaN_3_) (Kitamura et al., 2014), a significant decrease (*^∗∗^P* < 0.01 versus the control) of Rb_1_ uptake into rBMEC was seen in the presence of the inhibitor phloretin that inhibits glucose transporters (GLUT1) (Carruthers et al., 2009). This result indicated that Rb_1_ uptake crossing rBMEC membranes was independent of ATP and was actively transported by GLUT1-mediated facilitated diffusion.

**FIGURE 4:**
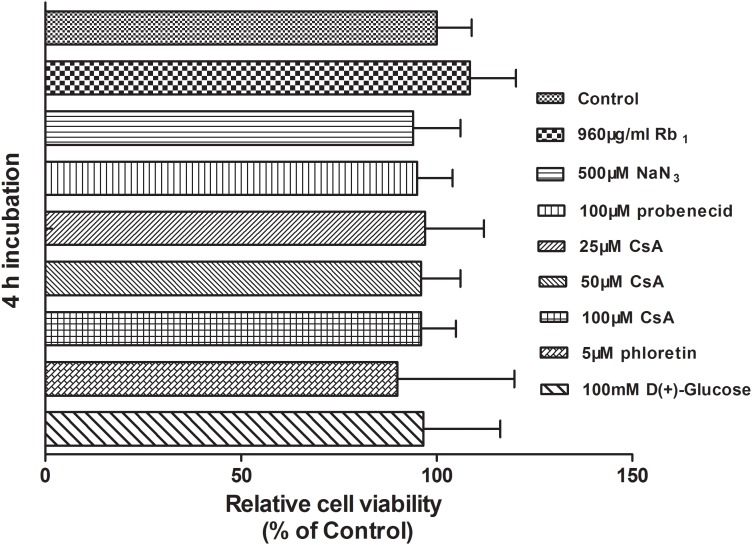
Cytotoxicity of test agents used in the study. The viability of rBMEC with a confluent monolayer exposure to the test agents used was examined by CCK-8 assay. Each column represents the mean ± SE of five replicates. Compared with the control, there was no statistically significant differences (*P* > 0.05).

In order to investigate whether the changes in Rb_1_ uptake by rBMEC in presence of the tested agents resulted from cell damage caused by the agents used, cells were incubated in Hanks’ solution containing of 500 μM NaN_3_, 100 μM probenecid, 25, 50, and 100 μM CsA, or 5 μM phloretin at 37°C for 4 h. Proliferation of rBMEC exposure to the test agents used was measured by CCK-8 assay. Compared with the control, no statistically significant differences existed (*P* > 0.05 versus the control, **Figure 4**). Thus, we concluded that the agents used did not have cytotoxicity at the concentration used, and that changes in the uptake of Rb_1_ did not result from cell damage. Phloretin, which significantly inhibited the uptake of Rb_1_ by rBMEC, was selected for further study.

### Inhibition of Phloretin on Rb_1_ Uptake Into rBMEC

**FIGURE 5:**
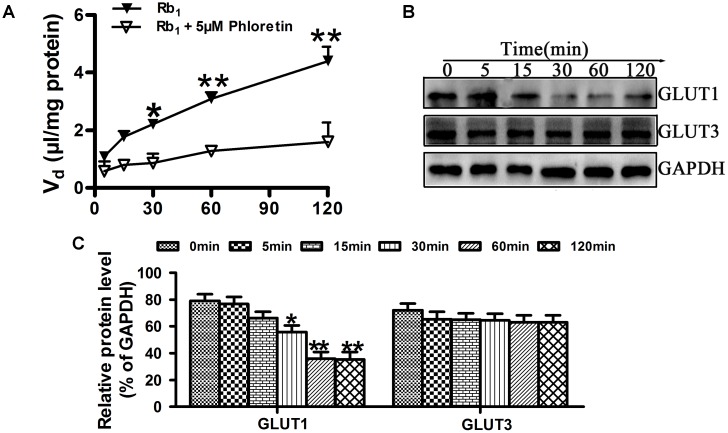
Effect of phloretin on Rb_1_ uptake into rBMEC. **(A)** To assess the roles played by GLUT1 in the transport of Rb_1_, the cellular uptake amount of Rb_1_ in confluent monolayers of rBMEC in 6-well plates was measured with 60 μg/mL Rb_1_ alone, or together with 5 μM phloretin at the designated time intervals at 37°C. Each point represents the mean ± SE (*n* = 3). Asterisks show a significant difference, ^∗^*P* < 0.05 and ^∗∗^*P* < 0.01 versus GLUT1 inhibitor groups. **(B)** Western blot assay was performed to evaluate the expression level of GLIUT1 and GLUT3 in rBMEC treated together with phloretin and Rb_1_ at the designated time intervals. **(C)** Relative protein level was quantitated using Image J. Each point represents as the mean ± SE (*n* = 3), with significant differences from control shown as ^∗^*P* < 0.05 and ^∗∗^*P* < 0.01, GLUT1 inhibitor groups versus control, using GAPDH as an internal control for protein loading.

In order to further investigate the effect of phloretin on Rb_1_ uptake into rBMEC, the time course of Rb_1_ uptake in the absence or presence of 5 μM phloretin was evaluated at 37°C. As shown in **Figure 5A**, the cell-to-medium (C/M) ratio of Rb_1_ was increased with time, but it was found that phloretin significantly decreased the C/M ratio of Rb_1_, especially from min 30 to 120. GLUT1 and GLUT3, encoded by the SLC2A1 and SLC2A3 gene, respectively, are 2 of 14 facilitative GLUT transporter members and are mainly expressed in CNS. To see whether the decreased C/M ratio of Rb_1_ was correlated with the expression level of GLUT1 or GLUT3 in rBMEC, western blot assay was performed (**Figure 5B**). Statistical analysis showed that the expression levels of GLUT1 at 30, 60, and 120 min were significantly lower than that of control (**Figure 5C**, ^∗^*P* < 0.05 and ^∗∗^*P* < 0.01 versus the control). However, compared with the control, there was no significant difference in the expression level of GLUT3. These data indicated that the decreased C/M ratio of Rb_1_ was correlative well to the down-regulated function and level of GLUT1 in rBMEC.

### Competitive Inhibition of D-Glucose on Rb_1_ Uptake by rBMEC

**FIGURE 6:**
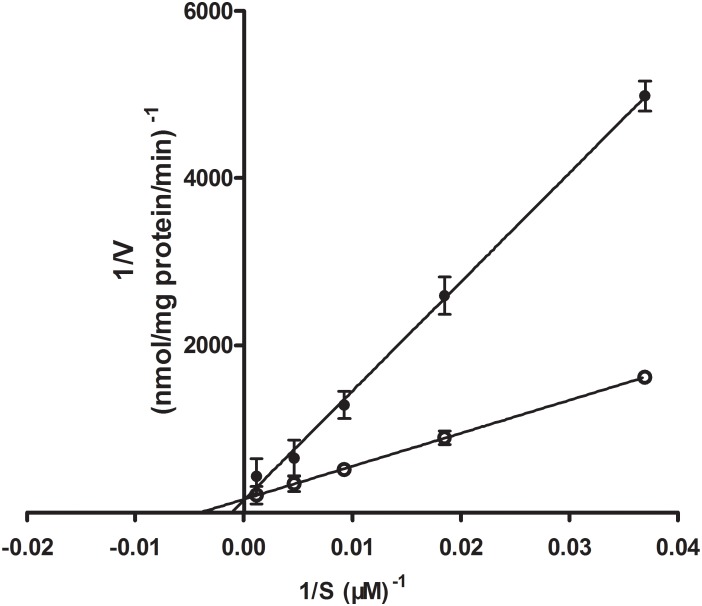
Lineweaver–Burk plot of the inhibitory effect of D-glucose on Rb_1_ uptake into rBMEC. Uptake profile of Rb_1_ (30–960 μg/mL) was measured in the absence (white marker) or presence of 100 mM D-glucose (black marker) at 37°C for 120 min. Each point represents the mean ± SE (*n* = 3).

While GLUT1 is able to transport galactose, mannose, and glucosamine, its principal physiological substrate is glucose. Under normal physiological conditions, the brain is absolutely dependent on glucose as a fuel source. GLUT1, as the major GLUT isoform expressed in brain endothelial cells, plays an important role in glucose transport across the BBB (Yeh et al., 2008). In the initial experiments, we analyzed the uptake characteristics of Rb_1_ by rBMEC. When we added the inhibitor phloretin of GLUT1, we observed significantly reduced C/M ratio of Rb_1_ uptake into rBMEC. Likewise, in Lineweaver–Burk plot of the inhibitory effects on the uptake of Rb_1_, the plots of Rb_1_ uptake in the absence and presence of D-glucose intersected at the ordinate axis (**Figure 6**), indicating that D-glucose competitively inhibited Rb_1_ uptake with a *K*_i_ value of 885 μM.

### Effects of Phloretin on Distribution of Rb_1_ in Plasma and Brain of Normal Rats

**FIGURE 7:**
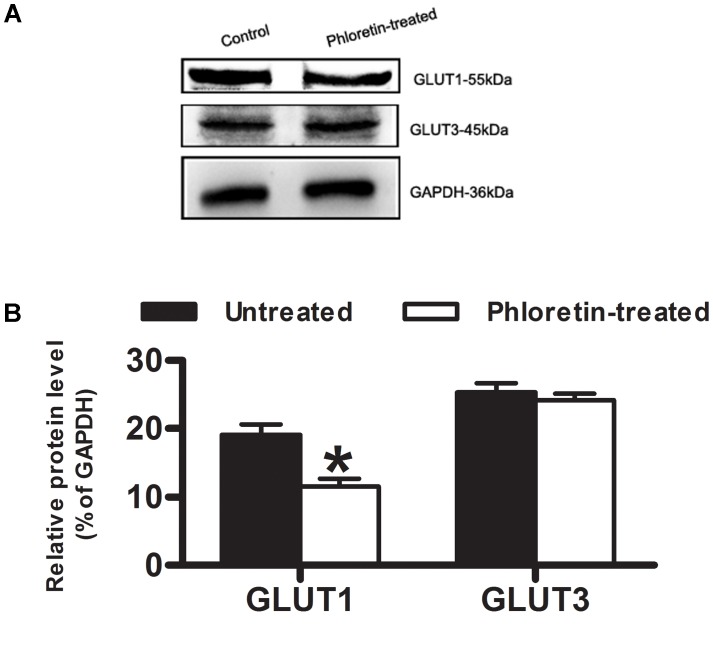
Expression of GLUT1 and GLUT3 in normal rat brain. **(A)** Western blot assay was to estimate whether the decreased brain–plasma concentration ratio of Rb_1_ was correlated with the expression level of GLUT1 or GLUT3 in normal rat brain. **(B)** Relative protein level was quantitated using Image J. GAPDH was stained as an internal control for protein loading. Each column represents the mean ± SE (*n* = 3), with significant differences from the phloretin-untreated control groups shown as ^∗^*P* < 0.05.

Except for the time point of 0.5 h after a single *i.v.* administration of Rb_1_ to the normal rats given phloretin for consecutive 1 week, plasma concentrations of Rb_1_ in the phloretin-treated normal rats at 2 h and 6 h were no statistically significant differences than those in the untreated normal rats. Whereas cerebral concentrations of Rb_1_ in the phloretin-treated normal rats were significantly lower than those in the untreated normal rats (**Table 1**). As a result, the brain-to-plasma concentration ratio of Rb_1_ in the phloretin-treated normal rats appeared to be significantly lower than that in the untreated normal rats, indicating that phloretin inhibits the transport of Rb_1_ across the BBB. To see whether the decreased brain–plasma concentration ratio of Rb_1_ was also related to the expression level change of GLUT1 or GLUT3 in the phloretin-treated normal rats, western blot assay was performed. The western blot results further showed that the expression levels of GLUT1 in the brain of normal rats given phloretin for 1 week were significantly decreased than those in the untreated normal rats (*P* < 0.05, **Figure 7**). Although phloretin also binds to the GLUT3, it potently inhibits the GLUT1-type glucose transporter (Martin et al., 2003). These results demonstrated that the decreased brain–plasma concentration ratio of Rb_1_ was correlated well with the decreased function and level of GLUT1 in the phloretin-treated normal rats, conforming the involvement of GLUT1 in Rb_1_ transport across the BBB into brain.

**Table 1:** Effect of phloretin on Rb_1_ distribution in plasma and brain of normal rats.

	0.5 h		2 h		6 h	
			
	Untreated	Phloretin-treated	Untreated	Phloretin-treated	Untreated	Phloretin-treated
Plasma concentration (μg/mL)	69.33 ± 4.5	48.7 ± 2.9**	39.7 ± 4.1	33.2 ± 1.4	35.2 ± 3.0	27.0 ± 1.5
Brain concentration (ng/g brain)	489.6 ± 58.3	105.1 ± 15.1*	193.8 ± 11.1	84.8 ± 4.1**	114.2 ± 24.0	39.9 ± 4.9*
Brain-to-plasma concentration ratio (*K*_p_, μL/g brain)	7.09 ± 1.01	2.15 ± 0.19*	4.9 ± 0.35	2.56 ± 0.06*	3.24 ± 0.53	1.59 ± 0.005*


*The rats were treated with phloretin (5 mg/kg/day, *i.v.*) for 1 week consecutively. The control rats only received saline. The Rb_*1*_ concentrations in plasma and brain were measured at 0.5, 2, and 6 h after injection of phloretin on day 7. Each value represents the mean ± SE (*n* = 6). Asterisks show a significant difference, ^∗^*P* < 0.05 and ^∗∗^*P <* 0.01 versus phloretin-untreated control groups.*

## Discussion

In the present study, Rb_1_ transport across the BBB was characterized by means of both *in vitro* uptake studies using primary rBMEC and *in vivo* brain–plasma concentration ratio (*K*_p_). Uptake of Rb_1_ by rBMEC was time- and temperature-dependent. The uptake of Rb_1_ increased linearly with time until 120 min, and a plateau of accumulation was observed at 120–240 min. However, the intracellular amounts of Rb_1_ at 4°C were lower than those at 37°C (**Figure 1**). Uptake of Rb_1_ was concentration-dependent, with *K*_m_ and *V*_max_ values of 119.2 μM and 0.0034 nmol/(mg protein ⋅ min), respectively. The saturable component (*V*_max_/*K*_m_) and the nonsaturable component (*P*_diff_) of Rb_1_ transport were calculated to be 0.03 and 0.0023 μL/(mg protein ⋅ min), respectively. Thus, 90% of the total Rb_1_ uptake is accounted for by the saturable component in the low concentration range. These results indicated that uptake of Rb_1_ into rBMEC was not solely driven by a simple passive diffusion process. In our *in vivo* study, the brain-to-plasma concentration ratios of ginsenoside Rb_1_ measured at 0.5, 2, and 6 h in untreated normal rats were significantly larger than that in phloretin-treated normal rats, suggesting carrier-mediated active uptake transport into brain across the BBB.

To further study the effects of transport systems with Rb_1_, other transporter inhibitors were used to evaluate transport activity of metabolic energy, Pgp, OATPs, and GLUT1. Pgp is one of the numbers of the ABC transporter superfamily, mediating the efflux from brain to blood and playing an import role *in vivo* to protect the brain from toxic compounds. OATPs play large roles in transporting endogenous molecules across cell membranes. GLUT1 is expressed in many cell types, but is at its highest level in the human erythrocyte membrane, through which glucose can freely keep in equilibrium between the serum and the red cell cytoplasm. And furthermore, GLUT1 is a major GLUT isoform expressed in cerebral endothelial cells, which plays an important role in cerebral glucose uptake. Cytochalasin B and phloretin would inhibit the GLUT1 activity (Koranyi et al., 1991; Simpson et al., 2001). Rb_1_ uptake by rBMEC was significantly decreased by phloretin, but was not inhibited by the inhibitors of metabolic energy (NaN_3_), P-gp (CsA), and OATPs (probenecid) (**Figure 3**). Although the GLUT1 can be capable of transporting galactose, mannose, and glucosamine, its principle physiological substrate is D-glucose. The *K*_i_ value for GLUT proteins is the concentration of blood glucose at which delivery into the cell, and GLUT1 is a high-affinity glucose transporter with a *K*_i_ for glucose of around 1 mM (Burant and Bell, 1992). In our study, the uptake of Rb_1_ was competitively inhibited by D-glucose with a *K*_i_ value of 885 μM. Therefore, the results indicate that the transport of Rb_1_ across BBB is mainly mediated by GLUT1, although we cannot deny the possibility that Rb_1_ is transported by any other transporter(s).

In the inhibition experiments when we determined the effect of phloretin on Rb_1_ uptake into rBMEC, we found a statistical decrease of the C/M ratio of Rb_1_ by phloretin after 30 min. Western blot assay showed that the decreased C/M ratio of Rb_1_ uptake into rBMEC was correlated well to the reduced level of GLUT1 in phloretin-treated rBMEC (**Figure 5**). These results are suggestive of a transporter-mediated uptake of Rb_1_ into rBMEC, possibly via GLUT1. This concept was further supported by evidence that the decreased brain-to-plasma concentration ratio of Rb_1_ was also correlative well with the down-regulated function and level of GLUT1 in phloretin-treated normal rats. The brain-to-plasma concentration ratio of a drug represents the degree of a drug’s penetration into brain across BBB. In the study, it was found that the brain–plasma concentration ratios of Rb_1_ in the phloretin-treated normal rats were significantly lower than those in the phloretin-untreated normal rats. And the expression level of GLUT1 in the phloretin-treated normal rats was down-regulated in comparison with the untreated control rats. However, we found the decreased level of GLUT3 in the phloretin-treated normal rats. Phloretin is the potent inhibitor of GLUT1-type glucose transporter although it is not highly selective and also binds to other GLUT isoforms (Martin et al., 2003). Thus, we may conclude that GLUT1 is involved in the Rb_1_ transport across the BBB into brain from circulating blood.

Due to a facilitated uptake of Rb_1_ into rBMEC, it can be inferred that Rb_1_ transport will be also observed in other cells that express GLUT1. GLUT1 is a 492-amino acid residues protein. It is expressed in most tissues at its highest level in fetal tissues (Zhao and Keating, 2007). The highest levels in adult humans are found in cerebral microvessels, colon, and kidney, but very low in skeletal muscle and liver (Gould and Bell, 1990). Some researchers have found that GLUT1 has a significant effect on the survival of the pre-implantation embryo (Moley, 2001). GLUT1 is also found in astrocytes, which has been considered to supply glycolytically derived lactate to neurons for a major energy source (Pellerin et al., 2002). The erythrocyte-type transporter protein GLUT1 has been also extensively investigated. And, this is possible that the transport in or on erythrocytes promotes an efficient compound exchange between the red cell and the capillary endothelium (Highley and De Bruijn, 1996). The meaning of uptake of drugs by erythrocytes has been specifically reported in the early stages (Highley and De Bruijn, 1996; Hinderling, 1997). The distribution of some molecules into erythrocytes can promote their storage, transport, and metabolism, and then may affect their activity (Kurlbaum et al., 2013). Because of erythrocytes consisting of a transport system with high capacity and low affinity compared to plasma proteins, the elimination of half-life of substances from different blood constituents might differ and the discharge from erythrocytes is often faster than the loss from plasma proteins (Schrijvers, 2003). Therefore, our future study will focus on the uptake of Rb_1_ by red blood cells, further confirming the involvement of GLUT1 in Rb_1_ transport across cell membranes.

Our study has a number of limitations. The inhibition experiments were done with Rb_1_ in the presence of various transporter inhibitors. Phloretin, an inhibitor of GLUT1, also interacts with other transporters such as the monocarboxylate transporter (Nagase et al., 2014), sodium glucose co-transporter SGLT-1 (Raja et al., 2003), volume-sensitive chloride channels (Fan et al., 2001), aquaporin water channels (Moshelion et al., 2004), or urea transporter (Konno et al., 2007). However, it reminds us that the significant decreased uptake of Rb_1_ by rBMEC in the presence of phloretin and D-glucose supports our point of view that uptake of Rb_1_ by rBMEC is an active transport process. Yet it cannot be excluded that additional transport processes play a major role as it was proposed by Sugano et al. (2010) that carrier-mediated and passive transport processes can coexist. Finally, we did not study whether the glucose influx in rBMEC was influenced by Rb_1_ or the precise type of interaction with the GLUT1 transporter. The type of interaction with the GLUT1 seems to be complicated as compounds can behave as competitive or noncompetitive inhibitors regarding glucose uptake or exit while the transporter has binding sites for phloretin (Kurlbaum et al., 2013). Although we have no further details on Rb_1_ transport, we discovered that it was the GLUT1 that involved in Rb_1_ transport across BBB.

To summarize, our present results indicate that the uptake of Rb_1_ into rBMEC is diminished in the presence of phloretin and D-glucose, suggesting that it is actively taken up into rBMEC, possibly via GLUT1. *In vivo* study using phloretin treatment suggests that GLUT1 plays a major role in active transport of Rb_1_ across BBB into brain from circulating blood.

## Author Contributions

X-YL and Q-ZL conceived and designed the experiments. Y-ZW, YL, and YJ performed the experiments. Y-ZW, QX, and WW analyzed the data. Q-QC contributed reagents/materials/analysis tools. Y-ZW and X-YL wrote the paper.

## Conflict of Interest Statement

The authors declare that the research was conducted in the absence of any commercial or financial relationships that could be construed as a potential conflict of interest.
